# Manifestation of epicardial type 1 electrocardiogram pattern with temperature elevation during open chest surgery in a patient with Brugada syndrome

**DOI:** 10.1016/j.hrcr.2022.07.003

**Published:** 2022-07-11

**Authors:** Akihito Ideishi, Satoshi Nagase, Naonori Kawamoto, Satsuki Fukushima, Tomoyuki Fujita, Kengo Kusano

**Affiliations:** ∗ Division of Arrhythmia, Department of Cardiovascular Medicine, National Cerebral and Cardiovascular Center, Suita, Japan; † Department of Advanced Arrhythmia and Translational Medical Science, National Cerebral and Cardiovascular Center, Suita, Japan; ‡ Department of Cardiovascular Surgery, National Cerebral and Cardiovascular Center, Suita, Japan

**Keywords:** Brugada syndrome, Fever, Type 1 electrocardiogram, Temperature elevation, Unipolar electrogram, Epicardium, Mapping, Ablation

**Key Teaching Points**

• In Brugada syndrome, epicardial unipolar and bipolar electrogram recording with temperature elevation and pilsicainide administration can unmask and highlight arrhythmia substrate.
• Type 1 pattern with epicardial unipolar recording on the right ventricular outflow tract was demonstrated by temperature elevation concomitant with QT shortening, although demonstrated by pilsicainide administration concomitant with QT prolongation.
• Temperature elevation and sodium channel blocker administration may unmask and exaggerate the arrhythmogenic substrate in Brugada syndrome by different mechanisms.

## Introduction

Brugada syndrome (BrS) is characterized by right precordial J-ST segment elevation on electrocardiogram (ECG) and sudden cardiac death from ventricular fibrillation (VF).^1^ Provocation with a sodium channel blocker (SCB) is effective in unmasking BrS. However, this is not a physiological response and can be iatrogenic. Several reports have shown that fever can unmask BrS-type ECG and occasionally cause specific premature ventricular contractions followed by spontaneous VF.^2^, ^3^, ^4^ Thus, understanding the fever-mediated electrophysiologic characteristics will be critical in evaluating the arrhythmia substrate in BrS. Administration of SCB or temperature elevation by warm saline infusion during epicardial mapping has already been reported on.^5^ However, there are no reports of both tests being performed consecutively in the same patient. Recent papers have suggested the significance of unipolar electrogram recordings during epicardial mapping.^6^, ^7^, ^8^, ^9^ However, the response of unipolar electrograms to temperature elevation has not yet been investigated. We report a case of BrS in which unipolar and bipolar electrograms with pilsicainide and a temperature elevation test were performed during open chest surgery.

## Case report

**Figure 1:**
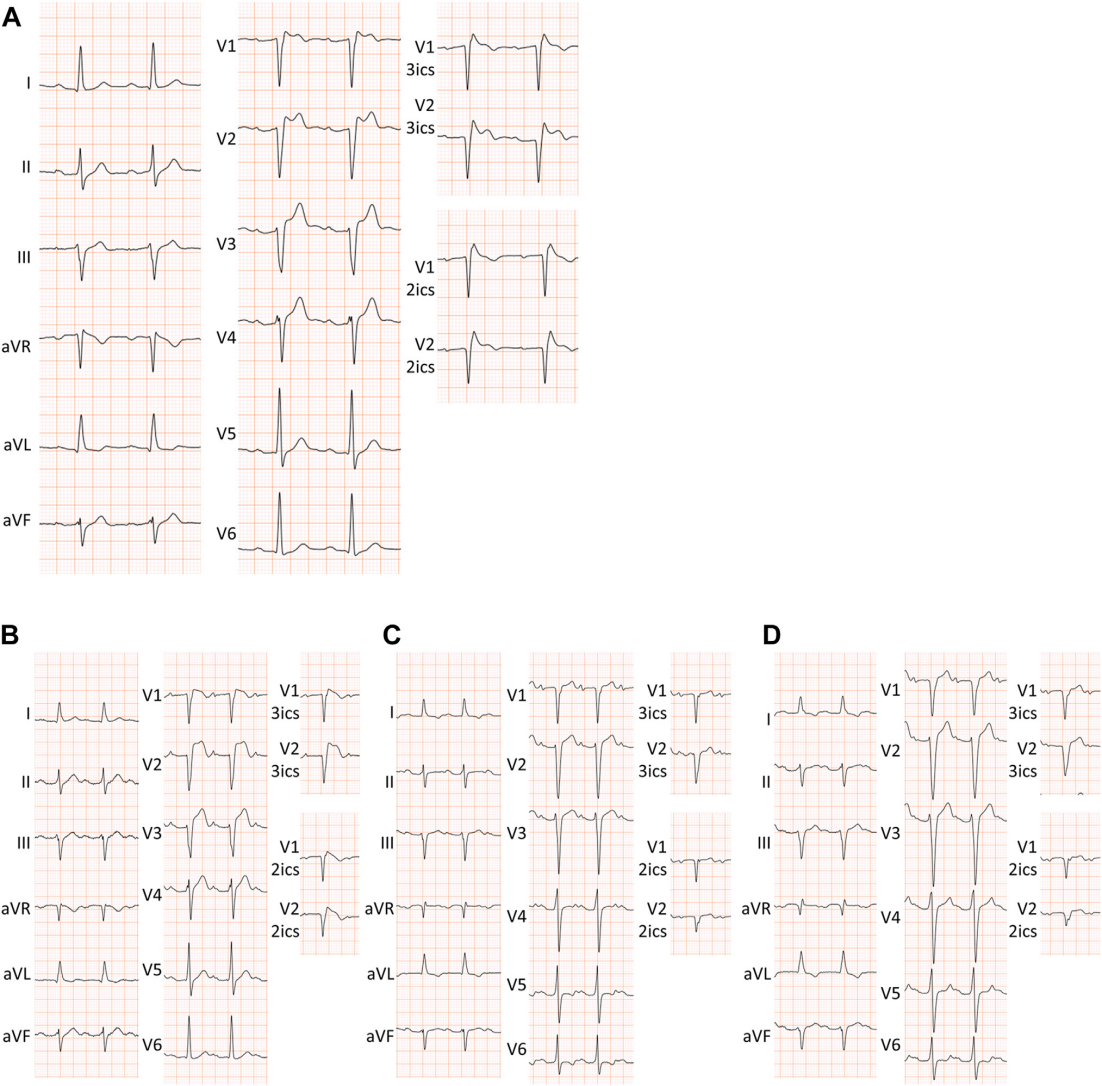
Electrocardiograms (ECG). **A:** ECG recorded in the chronic phase after implanted cardioverter-defibrillator implantation, when the patient was 70 years old. ECGs recorded more than 10 years ago were already discarded and no longer available. Type 1 ECG was present in leads V_1_–V_3_ics, V_1_–V_2_ics, and V_2_–V_2_ics. **B:** ECG on admission. Type 1 ECG was present in leads V_1_, V_1_–V_3_ics, V_1_–V_2_ics, and V_2_–V_2_ics. **C:** ECG after cryoablation. Type 1 ECG was not present, even in upper intercostal spaces. **D:** ECG after cryoablation and after pilsicainide administration. Type 1 ECG was not present. ics = intercostal space.

**Supplemental Figure 1:**
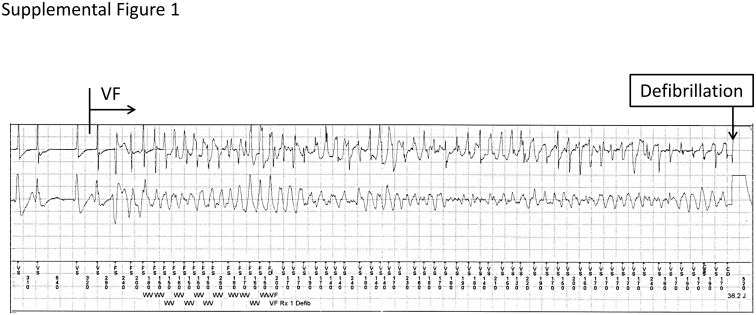
Implanted cardioverter-defibrillator interrogation demonstrating electrograms of ventricular fibrillation (VF) episode and appropriate device defibrillation.

A 76-year-old man was admitted to our institution because of spontaneous VF accompanied by implanted cardioverter-defibrillator (ICD) discharge confirmed by ICD interrogation (Supplemental Figure 1). This VF episode occurred almost at rest after light exertion around 3:00 PM, without any particular chest pain. Because of an unknown syncope attack, normal coronary arteries, no obvious abnormalities on echocardiography, spontaneous type 1 Brugada-pattern ECG (Figure 1A), and inducible VF in an electrophysiological study, the ICD had been implanted about 20 years prior to this hospitalization. Prolonged PR interval has always been noted in the past, but its cause is unknown. Genetic analysis revealed no concerning *SCN5A* variants. No ventricular tachyarrhythmia had been detected since the implantation of the ICD.

Despite normal echocardiography, coronary angiography revealed triple-vessel disease after admission and coronary bypass surgery was planned. Since type 1 ECG was still present (Figure 1B), epicardial ablation was also scheduled to be performed at the same time as the bypass surgery. Off-pump coronary artery bypass grafting was performed as follows: left internal thoracic artery to left anterior descending coronary artery; right internal thoracic artery to radial artery obtuse marginal branch, posterolateral branch, and atrioventricular node branch.

**Figure 2:**
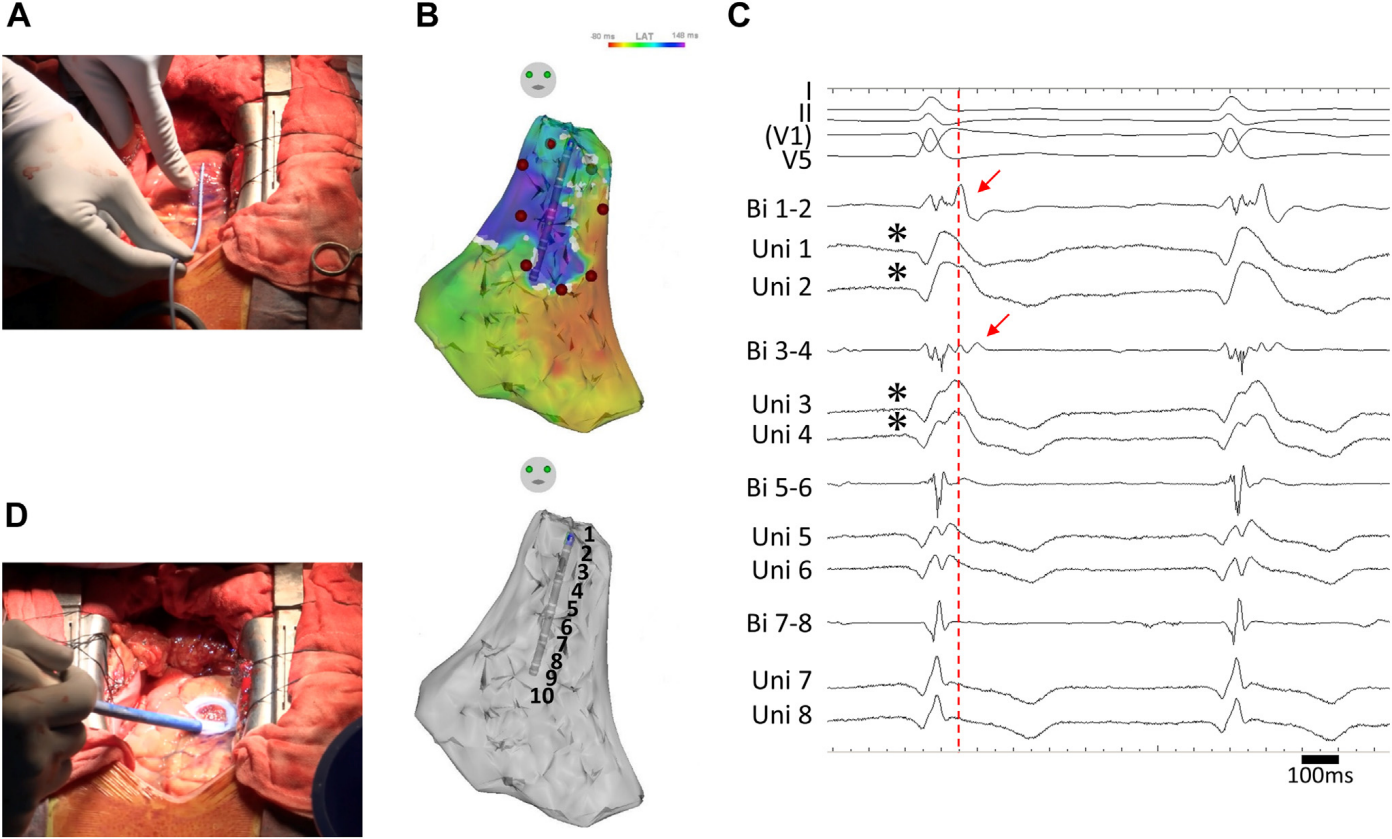
**A:** Epicardial mapping during open chest surgery. **B:** The upper figure shows an isochronal late activation map on the right ventricular (RV) epicardium during sinus rhythm. Cryoablation was performed in the area circled by the red tag, avoiding the coronary arteries. The lower figure shows the catheter position during the electrogram recording shown in Figures 2C and 3. **C:** Bipolar (Bi 1–2 to 7–8) and unipolar (Uni 1–8) epicardial electrograms on the RV outflow tract. Asterisks (∗) indicate unipolar type 1 pattern and red arrows indicate bipolar delayed potential. The gradient of a prominent J wave between the 2 unipolar electrograms is the cause of the delayed abnormal potential, as also reported by Haïssaguerre.^7^ Owing to the open chest surgery, the V_1_ lead was located outside the normal position. **D:** Epicardial cryoablation during open chest surgery.

Epicardial mapping and ablation were performed via a median sternotomy under general anesthesia. A 3-dimensional mapping system (CARTO 3; Biosense Webster, Irvine, CA) and a multipolar mapping catheter (DECANAV; Biosense Webster) were used. An electrode catheter (Inquiry; St. Abbott Laboratories, Chicago, IL) was placed in the inferior vena cava via a femoral vein as an indifferent electrode to record a unipolar electrogram. We recorded local unipolar electrograms with a 0.05- to 100-Hz bandwidth and local bipolar electrograms with a 30- to 250-Hz bandwidth on a digital recording system (LabSystem PRO; Bard Electrophysiology, Lowell, MA), as described previously.^9^, ^10^, ^11^ Carefully avoiding contact with the coronary bypass grafts and their anastomotic sites, epicardial mapping mainly on the right ventricle was performed using the multipolar mapping catheter (Figure 2A). The temperature in the pericardial sac was 36°C. Isochronal late activation mapping during sinus rhythm revealed that the latest zone of activation was mainly on the right ventricular outflow tract (RVOT) concomitantly with delayed or fractionated potentials (Figure 2B and 2C).

**Figure 3:**
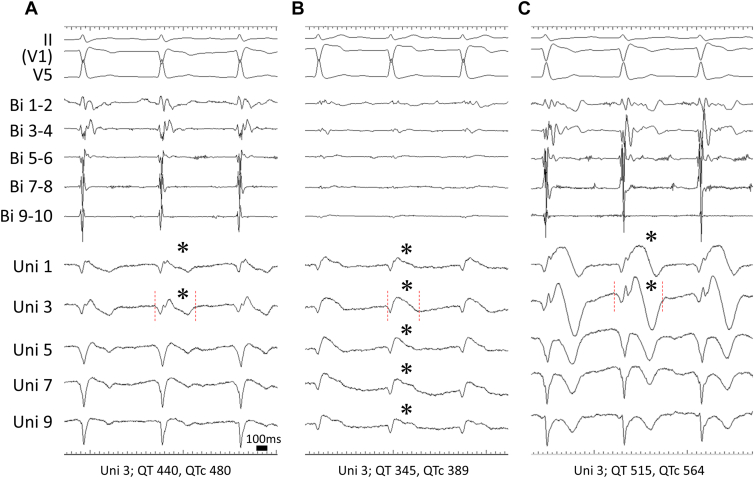
Electrocardiogram and epicardial electrograms at the right ventricular outflow tract. Bipolar (Bi 1–2 to 9–10) and unipolar (Uni 1–9) electrograms at the right ventricular epicardium before temperature elevation (**A**), after temperature elevation (**B**), and after pilsicainide administration (**C**). Asterisks (∗) indicate unipolar type 1 pattern. Red dotted lines indicate QT interval in Uni 3. As the temperature increased, the unipolar type 1 pattern appeared extensively and the amplitude of bipolar electrograms decreased markedly. After pilsicainide administration, fragmented and delayed potentials were exaggerated in the bipolar electrograms, and the unipolar type 1 pattern partially appeared. QT/QTc interval in unipolar leads shortened with temperature elevation; however, it was prolonged with pilsicainide administration.

After baseline mapping, the pericardial sac was completely filled with a sufficient volume of warm saline and maintained at 39°C–40°C to achieve local temperature elevation. After epicardial mapping mainly on the RVOT during temperature elevation, a 25 mg bolus of pilsicainide was administered intravenously after pericardial temperature returned to the previous state. The changes of bipolar and unipolar electrograms caused by warm saline injection and pilsicainide administration are shown in Figure 3. As the temperature increased, the amplitude of the bipolar electrograms decreased markedly, and the typical type 1 pattern appeared extensively in the unipolar electrograms. In contrast, pilsicainide administration caused a broad and pronounced delayed potential in the bipolar electrograms, and the type 1 pattern partially appeared in the unipolar electrograms. Epicardial cryoablation was performed on the RVOT and surrounding area showing delayed or fractionated bipolar potentials and type 1 unipolar pattern, carefully avoiding the bypass grafts with a surgical cryoablation probe (CryoICE; AtriCure, Mason, OH; Figure 2B and 2D). A total of 4 applications for 120 seconds at -60°C were delivered.

The postoperative period was uneventful and ECG at 3 weeks postsurgery showed disappearance of type 1 ECG even with recording on upper intercostal spaces and after pilsicainide administration (Figure 1C and 1D). The patient remained free of ventricular tachyarrhythmias until the 4-month-postoperative outpatient visit.

## Discussion

Fever is the most potent trigger to unmask and exaggerate type 1 Brugada ECG, which can lead to fatal ventricular tachyarrhythmias.^2^, ^3^, ^4^^,^^12^ However, the precise mechanism by which fever deteriorates arrhythmia substrate is still unclear. Administration of SCB and temperature elevation have been reported to reveal different ECG manifestations.^3^ In some patients with fever-induced type 1 ECG, SCB administration could not cause type 1 ECG. Temperature elevation–mediated ECG changes and electrophysiologic characteristics will be valuable in elucidating the arrhythmogenic substrate of type 1 ECG and spontaneous VF in BrS.

In this case, temperature elevation remarkably decreased ECG amplitude and a delayed component was present but indistinct in the bipolar electrogram on the RVOT. The typical type 1 pattern was widely visible in the unipolar electrogram, concomitant with QT shortening. In contrast, pilsicainide administration yielded pronounced fragmented/delayed potentials in the bipolar electrogram. However, the appearance of the typical type 1 pattern was partial and the QT interval was markedly prolonged in the unipolar electrogram. Although not definitive because full epicardial mapping was not performed owing to the time required for the bypass grafting, it appears that temperature elevation and SCB administration may cause different electrophysiological characteristics.

Boukens and colleagues^8^ have reported that epicardial J waves on unipolar recording are associated with late activation and are a therapeutic target for ablation. In agreement with their report, we also determined the ablation area by reference to the appearance of type 1 pattern on unipolar recording. The significance of bipolar and unipolar electrogram recordings during epicardial mapping, which demonstrate the correlation between depolarization and repolarization abnormalities, has also been reported recently.^6^^,^^7^^,^^10^^,^^11^ Previous basic studies also reported that elevating the temperature shortens the action potential duration in the epicardium, increases transmural dispersion of action potential duration, and causes J-ST segment elevation, leading to the development of reentrant tachyarrhythmia.^13^^,^^14^ The findings in this case are generally consistent with these previous reports.

There were several limitations to this study. Because open chest surgery was performed through a median sternotomy, right precordial ECG leads, including the upper intercostal spaces, could not be recorded during the procedure. Accordingly, correlation between surface ECG and epicardial unipolar electrogram was not assessed. In addition, epicardial mapping after cryoablation was not performed. Future studies are needed to verify whether the observations in this case are also found in other BrS patients.

## Conclusion

In this case with BrS, temperature elevation caused type 1 pattern and QT shortening on epicardial unipolar recording, while pilsicainide administration caused partial type 1 pattern with QT prolongation on the RVOT. Temperature elevation and administration of SCB may unmask and exaggerate the arrhythmogenic substrate in BrS by different mechanisms.
